# First Evidence of Coherent Bands of Strong Turbulent Layers Associated with High-Wavenumber Internal-Wave Shear in the Upstream Kuroshio

**DOI:** 10.1038/s41598-017-15167-1

**Published:** 2017-11-06

**Authors:** Takeyoshi Nagai, Daisuke Hasegawa, Takahiro Tanaka, Hirohiko Nakamura, Eisuke Tsutsumi, Ryuichiro Inoue, Toru Yamashiro

**Affiliations:** 1 Tokyo University of Marine Science and Technology, Department of Ocean Sciences, Tokyo, 108-8477 Japan; 2 Tohoku National Fisheries Research Institute, Japan Fisheries Research and Education Agency, Fisheries Oceanography and Resources Department, Shiogama, Miyagi 985-0001 Japan; 3 Kagoshima University, Faculty of Fisheries, Kagoshima, 890-0056 Japan; 4 Kyushu University, Research Institute for Applied Mechanics, Kasuga, Fukuoka 816-8580 Japan; 5 Research and Development Center for Global Change, Japan Agency for Marine-Earth Science and Technology, Yokosuka, 237-0061 Japan; 6 Graduate School of Science and Engineering, Kagoshima University, Kagoshima, 890-0065 Japan

## Abstract

The upstream Kuroshio flows through Okinawa Trough and the Tokara island chain, the region near the continental shelf of the East China Sea and shallow seamounts, where the Kuroshio can induce strong mixing over the shallow topography. Also, tidal currents over the rough topography may produce internal tides, and associated turbulence. The previous observations show energetic high vertical wavenumber near-inertial wave shear in the Kuroshio thermocline, which implies strong turbulent mixing. However, direct turbulence measurements in this region are very scarce. Using high lateral resolution (1–2 km) direct turbulence measurements, we show here, for the first time, that strong turbulent layers form spatially coherent banded structures with lateral scales of >*O*(10 km), associated with bands of near-inertial wave/diurnal internal tide shear of high vertical wavenumber in the upstream Kuroshio. The turbulent kinetic energy dissipation rates within these turbulent layers are >*O*(10^−7^ W kg^−1^), and estimated vertical eddy diffusivity shows >*O*(10^−4^ m^2^ s^−1^) on average. These results suggest that the high vertical wavenumber near-inertial waves propagating in the upstream Kuroshio could have large impacts on the watermass modifications, momentum mixing, nutrient supply, and associated biogeochemical responses in its downstream.

## Introduction

The Kuroshio, the western boundary current of the subtropical gyer in the North Pacific, has been known to transport large amounts of heat and salt from the tropical ocean^1^. Because the Kuroshio is inherently an ocean dynamic front, where the associated jet flows along the interface between seawaters of different water properties^2^, mixing processes in the Kuroshio Front play very important roles in watermass formation, transformation, and subduction^3–5^. In addition to this physical or hydrographical important role, the mixing in the Kuroshio has profound implications for biogeochemistry because the Kuroshio carries waters of relatively high nutrient concentrations from the tropics through its subsurface layers, as known as the nutrient stream^6,7^ similar to the Gulf Stream^8–11^. Earlier studies in the Gulf Stream^8,9^ and relatively recent studies in the Kuroshio^12,13^ have reported that elevated nutrient concentrations are found along these western boundary currents in their subsurface layers compared to that in the ambient waters of the same density. Because along-isopycnal stirring by mesoscale eddies tends to homogenize any tracer anomaly on the density surface in the ocean interior^14^, the elevated nutrient concentrations at the subsurface of the Kuroshio are most likely formed by across-density/diapycnal fluxes by irreversible diabatic turbulent diffusion. A previous study^9^ speculated that the turbulent diffusive nutrient flux by geostrophic vertical shear contributes to the concentration of elevated nutrients below the Gulf Stream. However, a limited number of direct turbulence observations at that time did not provide the supporting evidence for this hypothesis^15–17^.

On the other hand, the Kuroshio flows through shallow topography in its upstream along Okinawa Trough and near Tokara Strait, where the Kuroshio over the topography can induce strong turbulence and diapycnal mixing^18–20^. While a number of previous studies have reported that the beams of the M_2_ internal tide emanated from the rough topography induced strong turbulence in close proximity to canyons and seamounts^21,22^, recent shipboard and lowered Acoustic Doppler Current Profiler (ADCP) measurements in the upstream Kuroshio showed widespread large amplitude near-inertial internal wave shear of high vertical wavenumber in the Kuroshio and regions between continental margins and the Kuroshio^23^. A very recent field study in Tokara Strait reported near-inertial waves of even shorter vertical wavelengths ~50 m accompanied by pronounced turbulent dissipation rates of O(10^−7^–10^−6^ W kg^−1^)^20^. One of the most likely mechanisms to form the high vertical wavenumber shear is the near-inertial internal waves generated by the Kuroshio over shallow topography in Okinawa Trough and Tokara Strait^19^. Because the Kuroshio is forced to turn its direction from northeast to southeast when it approaches Kyushu island, high vertical wavenumber near-inertial internal waves could also be generated spontaneously by the meandering Kuroshio^24–28^. Furthermore, the upstream Kuroshio passes the so-called critical latitude, 28.9°N, where M_2_ internal tide energy can be efficiently converted to that of high vertical wavenumber near-inertial waves through parametric subharmonic instability (hereinafter PSI)^29,30^. Near the critical latitude, K_1_ or O_1_ diurnal internal tides generated over the rough topography are also near-inertial internal waves. In a strong baroclinic front, where geostrophic shear allows anomalously low frequency internal waves, a recent theoretical study^31^ showed that PSI of wind-driven near-inertial waves could generate high wavenumber internal waves with a frequency of half the local Coriolis frequency *f*/2. Regardless of their generation mechanism, these propagating near-inertial internal waves can be trapped on the anticyclonic side of the front where the lowest internal wave frequency is decreased by geostrophic lateral and vertical shears^32–34^. The trapped near-inertial waves can break into three-dimensional turbulence and enhance mixing of momentum and tracers^35–37^. However, direct turbulence measurements in the upstream Kuroshio are very scarce. Also, the lateral resolutions of previous turbulence measurements are not sufficient to resolve the coherent spatial patterns associated with high wavenumber near-inertial internal wave shear^15–17,38^. As a result, earlier studies did not find elevated turbulence in the thermocline of the Gulf Stream^15–17^, and concluded that turbulence in the ocean interior is caused by patchy random internal wave breaking, even in the Gulf Stream. In contrast, a number of recent studies have reported enhanced turbulent mixing in the thermocline of the Kuroshio^27,39–42^ and the Gulf Stream^43^ caused by propagating internal waves. However, the lateral resolutions of the turbulence data were still insufficient to provide direct evidence that the turbulence is caused coherently by banded internal-wave shear in the thermocline of the western boundary currents. Although a recent extensive microstructure time-series survey at low latitude^44,45^ reported that the strong turbulence coincided with the relatively large strain caused by wind-induced near-inertial waves of anomalously low frequency in the anticyclonic vorticity field^33^, it is not known if near-inertial internal waves form the spatially coherent strong turbulent layers in ocean fronts. In this study, a new turbulence and microstructure observation scheme is developed and utilized to measure turbulence directly with a high lateral resolution of 1–2 km in the upstream Kuroshio near Tokara Strait, where a large amount of energy from the Kuroshio can be dissipated. For the first time, the observations in this study suggest that propagating high vertical wavenumber near-inertial internal waves form spatially coherent banded layers of strong turbulence in the upstream Kuroshio, with *O*(100 m) and >*O*(10 km) vertical and lateral scales, respectively.

## Results

### Observations

**Figure 1:**
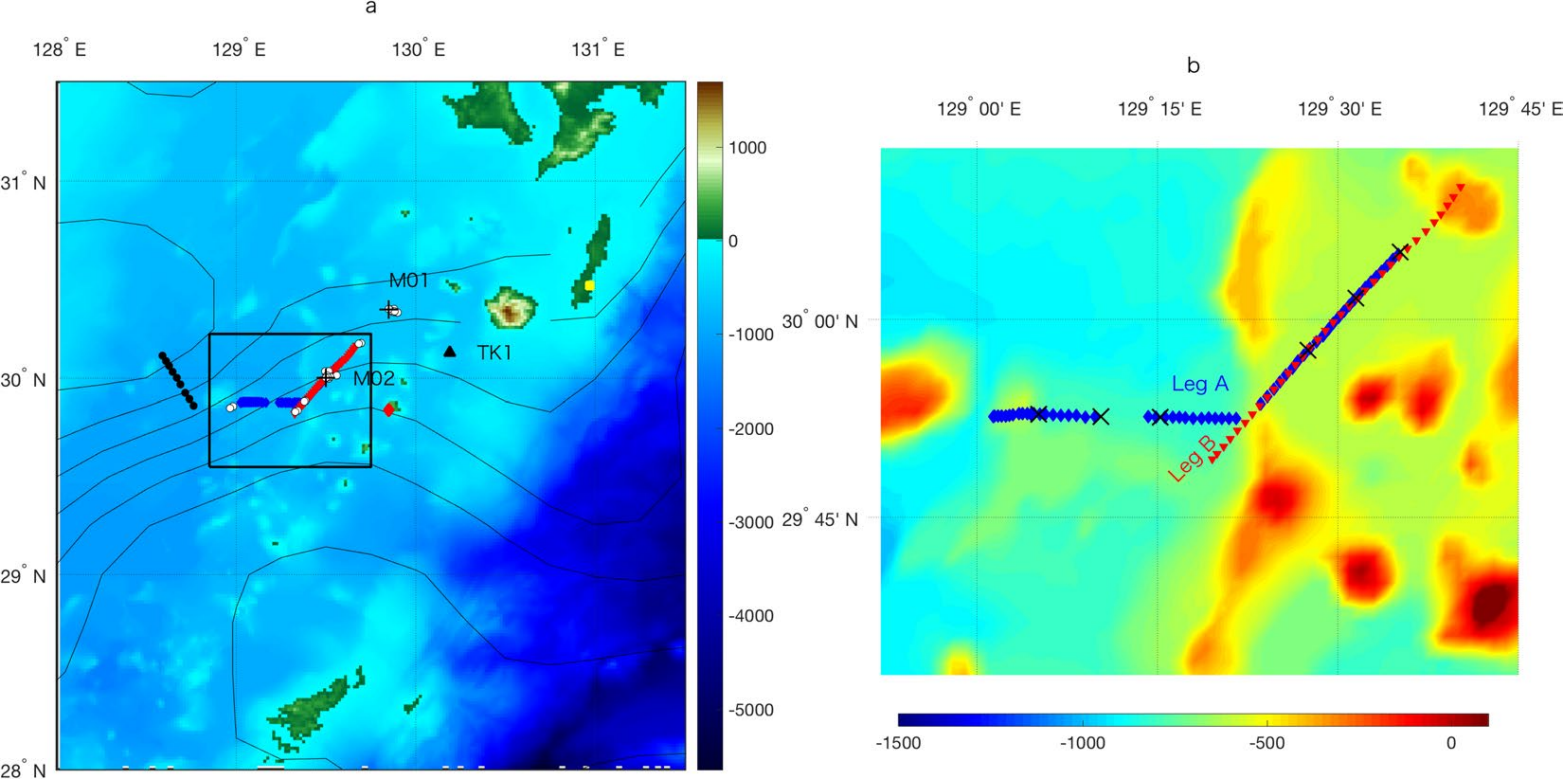
Observation site in Tokara Strait near the Tokara island chain off Kyushu island. (**a**) Underway-CTD (UCTD) observation line is shown as a thick black line, and Underway-VMP (UVMP) observation lines are in blue for Leg A and red for Leg B. Mooring stations Stn. M01 and M02 are indicated by black (+). CTD stations are denoted by solid white circles ($\circ$). Total number of the CTD profiles is 36, which consists of 13 profiles each at Stn. M01 and M02 during time series observations and 10 profiles at four other stations near the Kuroshio. The relatively long-term mooring Stn. TK1 is shown as a black triangle. Nakanoshima, and Tanegashima tidal stations are shown as a red diamond, and a yellow square, respectively. Black contours are AVISO sea surface height (m) averaged from November 14–19, 2016. (**b**) Enlarged map of the UVMP observation site. UVMP in Leg A is denoted by blue diamonds and Leg B by red triangles. Black crosses (×) indicate six XBT stations during Leg A. Color shadings indicate depth (m). The map is created with MATLB R2016b with ETOPO1 topography data available from NGDC NOAA https://www.ngdc.noaa.gov/mgg/global/.

The *in-situ* survey was conducted using the R/T/V *Kagoshima*-*maru* during November 12–20, 2016, near Tokara Strait where the upstream Kuroshio flows over the shallow seamounts and through the narrow straits near the Tokara island chain off Kyushu island (Fig. 1). Two mooring systems, which included an upward looking ADCP (75 kHz 20° beam angle Workhorse Long Ranger ADCP, Teledyne RDI) were deployed near the Kuroshio and measured horizontal currents and directions during November 13–19, 2016, at Stn. M01 (30.35°N, 129.85°E) and M02 (30.00°N, 129.50°E, Fig. 1a). Two transect observations were carried out to obtain current velocity data using a shipboard ADCP (75 kHz 30° beam angle, Ocean Surveyor, Teledyne RDI) and high-resolution towed microstructure data. The transect observations (hereinafter Leg A) were conducted on November 14 from west to east closely along the zonal direction for the first half of the transect, and continued in the northeast direction for the rest (thick blue line in Fig. 1b). The transect surveys were repeated (hereinafter Leg B; red in Fig. 1b) on November 18–19 close to the same ship track as for the second half of the Leg A observations. During these two transect observations, a new tow-yo Underway Vertical Microstructure Profiler (Underway-VMP, hereinafter UVMP; Rockland Scientific International, Victoria, Canada) was used in the Kuroshio Front to investigate high-resolution spatial structures of microscale turbulence. Prior to the transect observations of Legs A and B, the Underway Conductivity Temperature Depth profiler (hereinafter UCTD; Teledyne Oceanscience, California, USA) was deployed across front direction repeatedly (thick black line in Fig. 1a). Conductivity-Temperature-Depth (CTD) profiles also were made at 36 stations (Fig. 1a). Because the UVMP used in this study was a prototype with no conductivity sensor, simultaneously gathered salinity data are not available. However, because water properties obtained by these UCTD and CTD in the observed regions suggest a close relationship between temperature and density (Supplementary Fig. S1), we estimated density in Legs A and B from temperature alone (see Methods section). In addition to the mooring observations made during the shipboard surveys, the relatively long-term moored ADCP records from September 30, 2000 through July 1, 2001 obtained at Stn. TK1 (30.13°N, 130.19°E, Fig. 1a) in the vicinity of the shipboard observation sites were analyzed to investigate the mechanisms generating observed internal waves.

### High-vertical-wavenumber internal-wave shear

**Figure 2:**
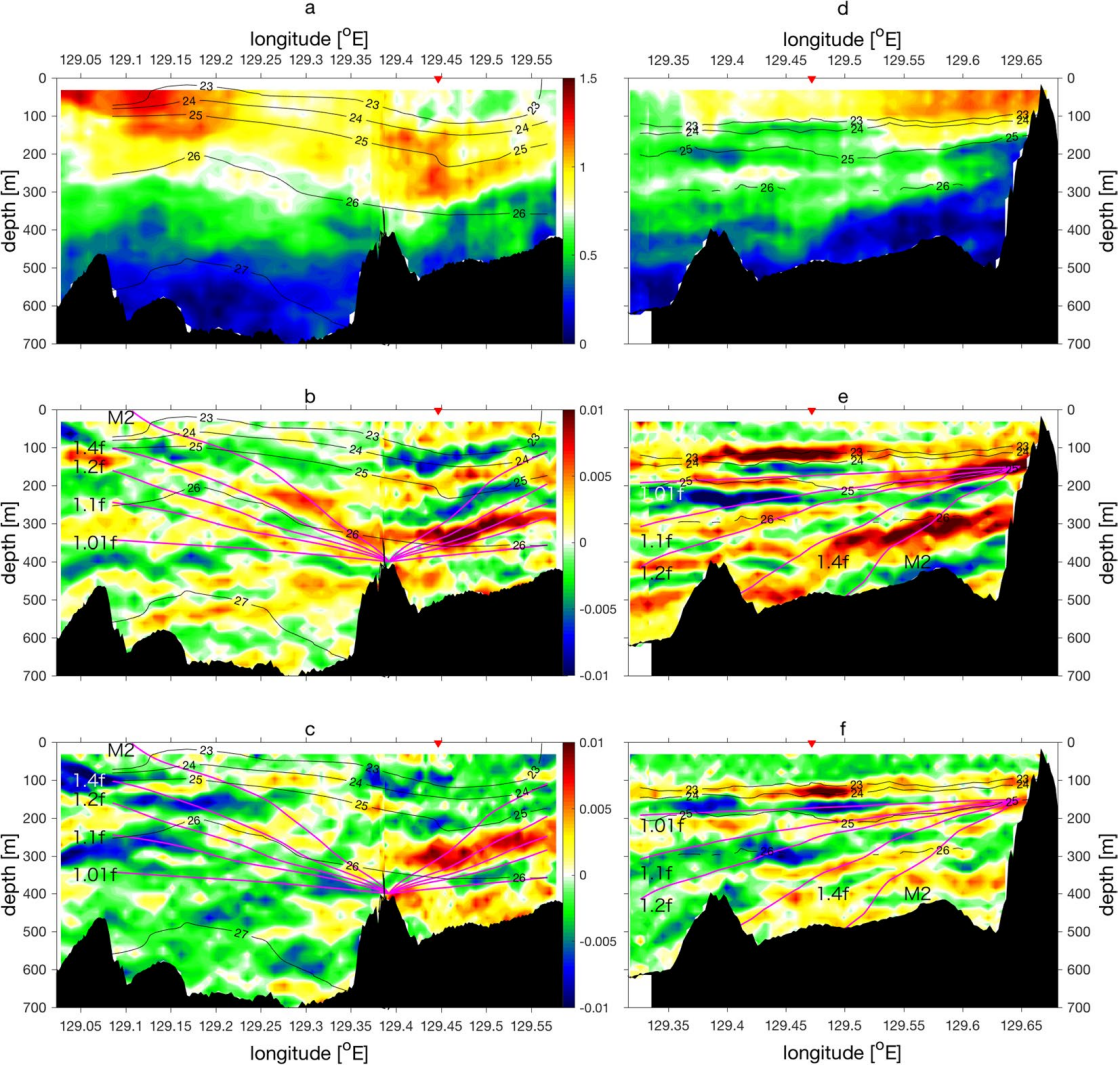
Vertical sections of the shipboard Acoustic Doppler Current Profiler velocity measurements. (**a**–**c**) are for Leg A (blue in Fig. 1b) and (**d**–**f**) are for Leg B (red in Fig. 1b). Horizontal absolute current velocity (m s^−1^) are shown in (**a**,**d**). Back-rotated shear [s^−1^] is shown for zonal shear *u*_*z*_(*t*_0_) (**b**,**e**), and for meridional shears *v*_*z*_(*t*_0_) (**c**,**f**) (see Methods section). Black contours are *σ*_*θ*_ (kg m^−3^). Magenta curves in (**b**,**c**,**e**,**f**) are internal-wave ray paths at frequencies of 1.01 *f*, 1.1 *f*, 1.2 *f*, 1.4 *f*, and M_2_ tidal frequency, where *f* is Coriolis frequency, as indicated in the panels. Red triangles in upper abscissa are the locations at which hodographs of shear are plotted for each Leg in Fig. 3.

**Figure 3:**
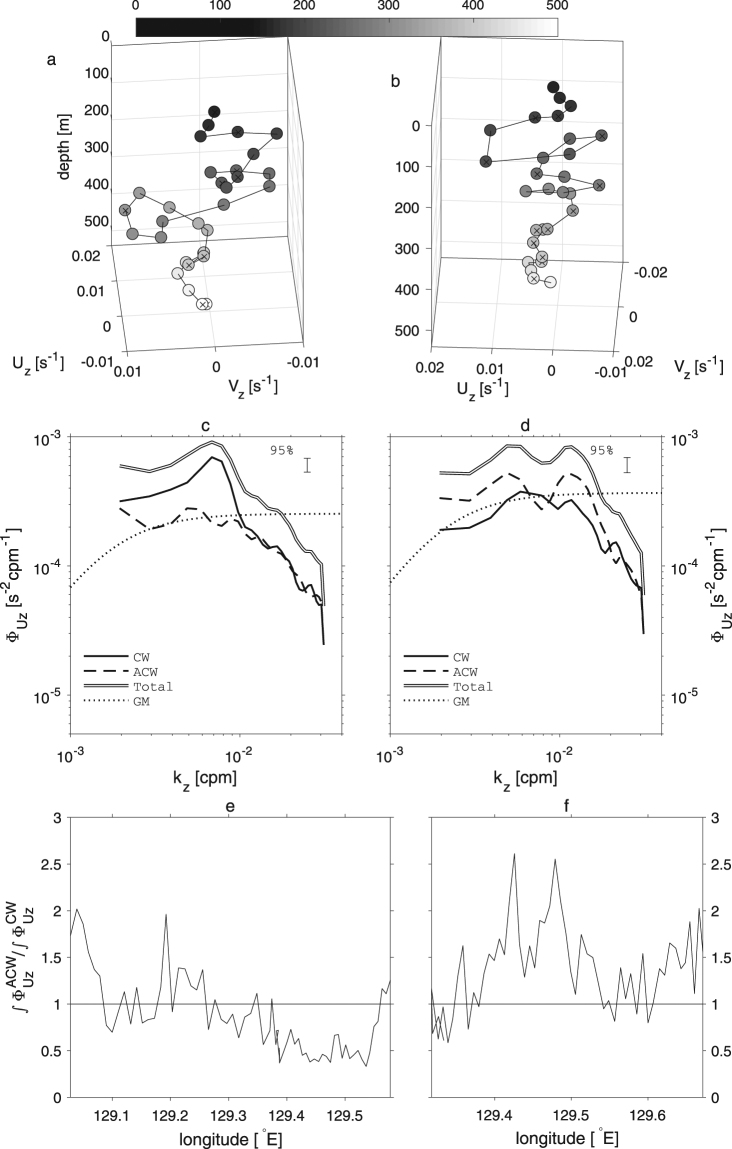
Hodographs of the back-rotated shear [s^−1^] are shown for (**a**) 129.447°E in Leg A (Fig. 2a–c) and for (**b**) 129.472°E in Leg B (Fig. 2d–f). Shading indicates depth (m). Average vertical wavenumber shear spectra [s^−2^ cp m^−1^] are shown for (**c**) Leg A and for (**d**) Leg B. Solid black lines (CW) are the clockwise rotating component with depth and dashed lines (ACW) are the anticlockwise rotating component. Solid black-white-black lines are for total shear spectra. Dotted lines (GM) are Garrett-Munk equilibrium internal wave shear spectra. (**e**,**f**) Ratio of integrated anticlockwise shear spectra to that for the clockwise component $\int {\varphi }_{ACW}^{{{\bf{u}}}_{z}}dk/\int {\varphi }_{CW}^{{{\bf{u}}}_{z}}dk$ (where ${\varphi }_{ACW}^{{{\bf{u}}}_{z}}$ and ${\varphi }_{CW}^{{{\bf{u}}}_{z}}$ are anticlockwise and clockwise components of shear spectrum, respectively and *k* is the vertical wavenumber) as a function of longitude in Leg A (**e**) and B (**f**).

Measured horizontal current velocity using the shipboard ADCP mostly shows a northeastward flow associated with the Kuroshio, consistent with the sea surface height (Fig. 1). During the Leg A observation, this flow was strongest in the westernmost edge of the transect, where the main stream of the Kuroshio flowed (Fig. 2a). On the other hand, in Leg B, the largest velocity magnitude was recorded at the northeastern part, the last half of the transect (Fig. 2d), as the continuous UVMP profiling in Leg B started from the southwestern region to the northeastern direction, reaching the main stream of the Kuroshio Front in the last quarter of the Leg B (Fig. 1). In both the Legs, current velocity magnitudes are largest near the surface, due to the baroclinic flow of the Kuroshio, with some exceptions where the stronger currents are found subsurface, especially on the anticyclonic side of the Kuroshio (Fig. 2a,d). Back-rotated velocity shear, which is the vertical gradient of the lateral velocity rotated horizontally, assuming the inertial rotation with respect to the reference time (see Methods section), shows bands of alternating positive and negative signs, close to the density surface (Fig. 2b,c,e,f), which are similar to a study by Rainville and Pinkel^23^. These banded shears are typical characteristics of the propagating inertia-gravity waves in a stratified ocean. The wavelengths of these banded shear structures are *O*(100 m) in vertical and *O*(10 km) in lateral direction, similar to Rainville and Pinkel’s study in this region^23^. With *λ*_*h*_ and *λ*_*v*_, horizontal and vertical wavelengths (m), respectively, $N=\sqrt{\partial b/\partial z}$ buoyancy frequency (rad s^−1^) (where *b* = −*gρ*/*ρ*_*o*_ is buoyancy (m s^−2^) with gravitational acceleration *g* (m s^−2^), seawater density *ρ* (kg m^−3^) and reference density *ρ*_*o*_ = 1025 (kg m^−3^), and vertical coordinate *z*(m)), and *f* Coriolis frequency (rad *s* ^−1^), the internal-wave dispersion relation, then implies that the frequency of these waves $\omega =\sqrt{({f}^{2}/{\lambda }_{z}^{2}+{N}^{2}/{\lambda }_{h}^{2})\,{\lambda }_{z}^{2}} \sim f$, based on the estimated *N*~*O*(10^−2^ rad s^−1^), and local *f*~*O*(10^−4^ rad s^−1^). The internal-wave ray paths computed, assuming a quiescent condition show that the angles of the internal wave rays with near-inertial frequencies are more consistent with the observed shear bands than those with the M_2_ tidal frequency (Fig. 2b,c,e,f). This also suggests that the observed high-wavenumber velocity shear is due to near-inertial internal waves. The total shear vertical wavenumber spectra averaged for the entire transect of Leg A and B suggest that shear variance is significantly larger than that of Garrett-Munk (GM) internal-wave equilibrium spectra^46,47^ by several factors, at vertical wavelengths larger than 100 m (Fig. 3c,d), at which the resolution of the shipboard ADCP starts to fail to resolve shear of shorter wavelengths. Computed Richardson numbers $Ri={N}^{2}/({u}_{z}^{2}+{v}_{z}^{2})$, where *u*_*z*_ and *v*_*z*_ are zonal and meridional shear, show relatively small values in the banded shear layers and near the surface. However, because of the coarse resolution of the ADCP shear, the *Ri* are mostly larger than the critical value for the Kelvin-Helmholtz instability *Ri* = 0.25 except near the surface and below 500 m in Leg A and 300 m in Leg B (Supplementary Fig. S2). The rotary shear spectra in Leg A show that the variance of clockwise rotating shear with depth is larger than that of anticlockwise rotating component (Fig. 3c), suggesting the dominant downward energy propagating internal waves. In Leg B, the clockwise and anticlockwise components of rotary shear spectra are comparable with slightly larger shear variance for the anticlockwise rotating component (Fig. 3d). The ratio of the integrated anticlockwise shear spectrum to that for the clockwise component $\int {\varphi }_{ACW}^{{{\bf{u}}}_{z}}dk/\int {\varphi }_{CW}^{{{\bf{u}}}_{z}}dk$ (where ${\varphi }_{ACW}^{{{\bf{u}}}_{z}}$ and ${\varphi }_{CW}^{{{\bf{u}}}_{z}}$ are anticlockwise and clockwise components of shear spectrum, respectively, and *k* is the vertical wavenumber) as a function of longitude suggests that large shear variance found at 129.3–129.575°E in Leg A is associated with the downward energy propagating internal waves, and that found at 129.4–129.55°E in Leg B is caused by upward energy propagating internal waves (Fig. 3e,f). The hodograph of the shear at 129.447°E in Leg A, where shear magnitude is large, shows that shear is rotating clockwise with depth, suggesting downward energy propagating internal waves, while that at 129.472°E in Leg B shows anticlockwise rotation for upward energy propagating internal waves, consistent with the trends found in the shear spectra (Fig. 3a,b,e,f). The estimated average internal wave energy is 6.4 J m^−3^ for Leg A, and 14.2 J m^−3^ for Leg B. The estimated mean group velocity, 3.5 × 10^−5^ m s^−1^ with the mean vertical wavelength of the internal wave, 150 m, results in the mean vertical energy flux of 0.2 mW m^−2^ for Leg A, and 0.5 mW m^−2^ for Leg B (see Methods section). These estimates are comparable to the rate of wind energy input to inertial motions computed by a slab model^48–50^ (see Methods section, Supplementary Fig. S3).

These results indicate that the high vertical wavenumber shear is caused by near-inertial internal waves propagating in the upward and downward directions in the Kuroshio near Tokara Strait. While the wind energy flux to the inertial currents is found to be sufficient to generate an observed amplitude of near-inertial waves, it is not clear how the observed high vertical wavenumber near-inertial internal waves are formed. We speculate that the possible mechanisms are (1) PSI of the M_2_ internal tide upstream, which was advected to the observation sites by the Kuroshio flow, (2) local PSI with anomalously lower minimum internal-wave frequency than *f* due to mean velocity shears, (3) K_1_ internal tides generated over rough topography, (4) near-inertial internal waves generated by the Kuroshio over shallow topography in Okinawa Trough and Tokara Strait, (5) spontaneously generated near inertial internal waves by the Kuroshio, and (6) PSI of wind-induced inertial waves to generate high wavenumber anomalously low frequency internal waves of half the Coriolis frequency, *f*/2 along isopycnal in a strong baroclinic front^31^.

### High-resolution tow-yo turbulence measurements

**Figure 4:**
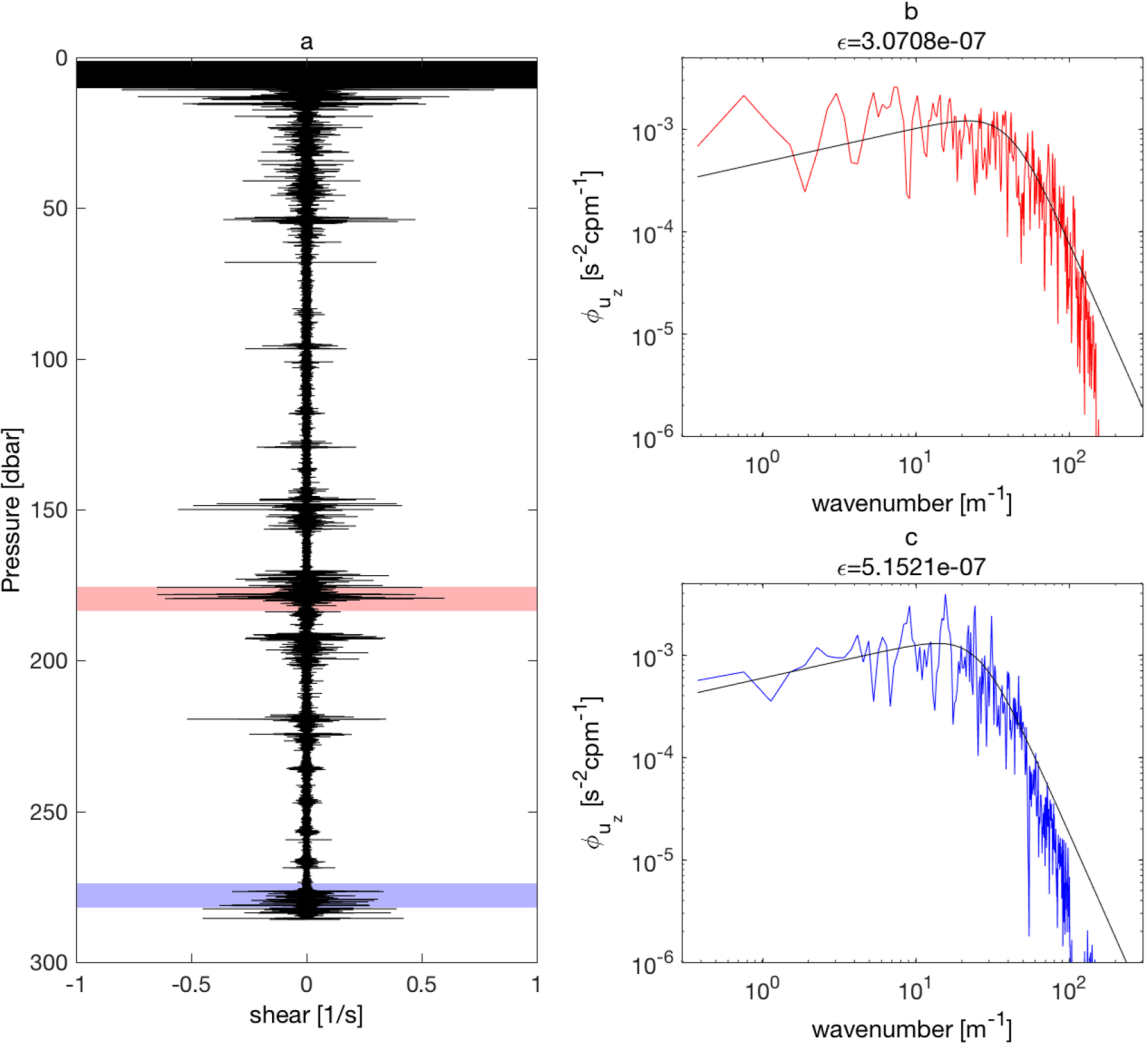
Microscale shear and shear spectra obtained from tow-yo observations. (**a**) One of the turbulent shear profile [s^−1^] and (**b**,**c**) turbulent shear spectra [s^−2^ cp m^−1^]. The shear spectra in red and blue correspond to the depth ranges indicated by the same color in (**a**). The Nasmyth empirical shear spectra are shown in (**b**,**c**) as black curves for each computed turbulent kinetic energy dissipation rate *ε* [W kg^−1^].

The shipboard ADCP measurement shows the large amplitude banded high vertical wavenumber shear, similar to previous studies^23^. Here, we show, for the first time, that these large amplitude near-inertial internal wave shears generate strong turbulence of spatially coherent banded structures, employing a newly developed high-resolution (1–2 km) tow-yo microstructure profiling technique, using 1.5 mm dyneema rope. The thin rope allows the microstructure profiler (VMP-250) to sink relatively smoothly with small drag down to 300 m depth. The measured turbulent shear spectra by the tow-yo profiling show overall good agreement with the Nasmyth empirical shear spectrum^51^ (Fig. 4). This novel technique, therefore, allows us to resolve spatial structures of intermittent turbulence, which have been unrecognized because of the coarse resolution measurements by the previous microstructure profiling.

**Figure 5:**
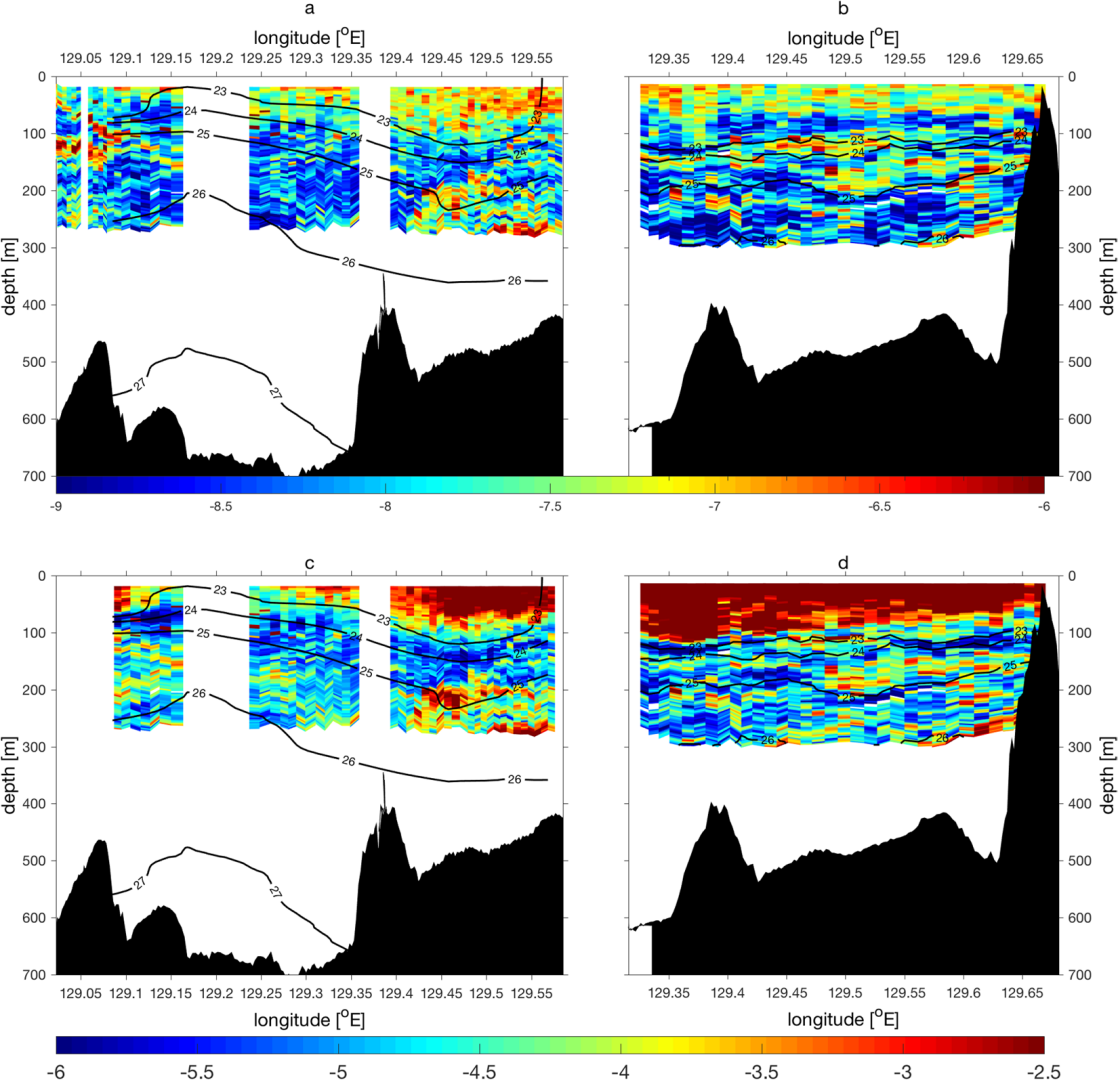
Vertical section of (**a**,**b**) measured turbulent kinetic energy dissipation rates ${{\rm{log}}}_{10}\,\varepsilon$ [W kg^−1^], and (**c**,**d**) estimated vertical turbulent eddy diffusivities ${{\rm{log}}}_{10}\,{K}_{\rho }$ [m^2^ s^−1^] for (**a**,**c**) Leg A and (**b**,**d**) Leg B. Contours are *σ*_*θ*_.

In the upper 100 m, the measured turbulent kinetic energy dissipation rate, *ε* is large, *O*(10^−7^ W kg^−1^), because of turbulence within the surface boundary layer, which is forced by momentum and heat fluxes at the surface (Fig. 5). In the stratified layers below 100 m depth, away from the surface boundary layer, the dissipation rates still frequently exceed *O*(10^−7^ W kg^−1^), and reach *O*(10^−6^ W kg^−1^) at some locations (Fig. 5a,b). These dissipation rates are 100 to1000 times greater than those found typically in the open water thermocline. More importantly, these large dissipation rates form coherent along-isopycnal bands of strong turbulent layers. These strong turbulent layers found beneath and on the less dense side of the Kuroshio correspond very well to the large amplitude near-inertial wave shear bands, suggesting that the observed near-inertial internal waves of high vertical wavenumber break into microscale turbulence. The comparison between our observed turbulent dissipation rates and previous internal-wave parameterization for energy dissipation rates^52,53^ show a positive correlation (r = 0.63, p-value = 0.0001) within the same orders of magnitude (Supplementary Fig. S4). For the first time, our high resolution microstructure surveys provide evidence that strong turbulent layers with spacial scales of *O*(100 m) and *O*(10 km) in the vertical and lateral directions, respectively, are coherently formed along the near-inertial internal-wave shear bands (Figs 2 and 5a,b). To investigate the significance of this turbulence not only for energy dissipation but also for the vertical mixing and diffusion, the vertical turbulent eddy diffusivity is estimated using measured dissipation rates *ε* and buoyancy frequency *N* with the previous diffusivity model^54^, namely,

1 $${K}_{\rho }=\gamma \frac{\varepsilon }{{N}^{2}},$$

where *γ* = 0.2 is a mixing efficiency factor^54^. The estimated vertical turbulent eddy diffusivities are >*O*(10^−2^ m^2^ s^−1^) within the surface boundary layer, which is not surprising considering continuous forcing at the surface with weak stratification in the surface boundary layer (Fig. 5c,d). The vertical eddy diffusivities in the stratified subsurface layers, however, are notably high, *O*(10^−4^ m^2^ s^−1^) on average, and >*O*(10^−3^ m^2^ s^−1^) at many locations below 100 m depth (Fig. 5c,d). The high diffusivity layers are structured in a banded form, reflecting the spatial structures found in the dissipation rates.

## Discussion

Early microstructure surveys in the 1970s–1980s did not find strong turbulence across the western boundary current, i.e., the Gulf Stream, but found intermittent turbulent patches due to random internal wave breaking^15–17^. Relatively recent microstructure measurements in the upstream Gulf Stream, Florida Current^38^, and in the downstream Gulf Stream^43^, reported the possibility of the internal wave induced turbulence, but whether turbulent layer is formed coherently with the banded internal wave shear is unclear due to the coarse lateral resolution of the surveys. In contrast, this study found coherent bands of very strong turbulence clearly associated with the large amplitude, high vertical wavenumber internal-wave shear, in the upstream Kuroshio.

**Figure 6:**
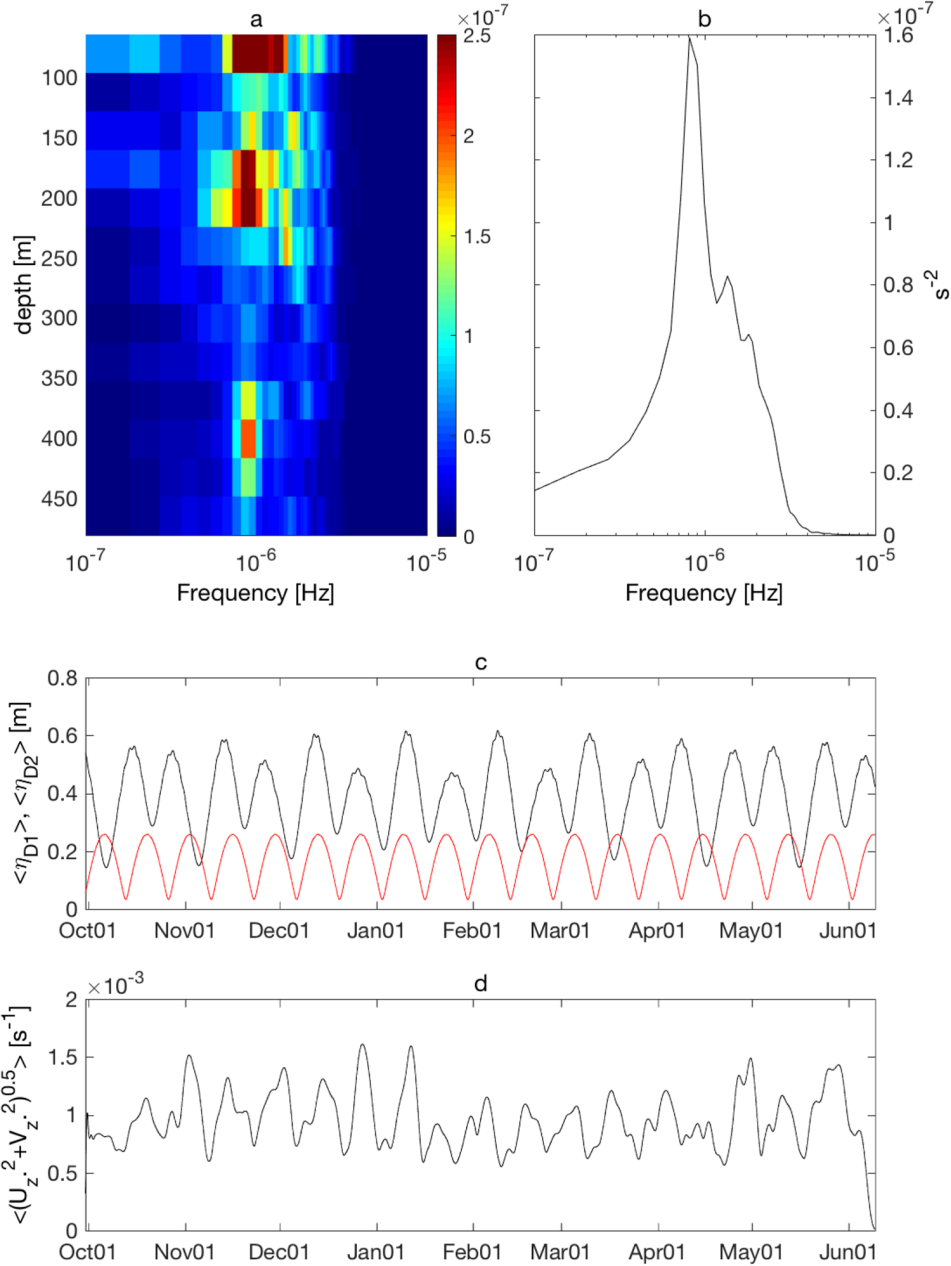
Long-term moored ADCP data analysis. The variance preserved spectra [s^−2^] of the 30-h lowpass near-inertial shear $\langle \sqrt{{u}_{z}^{2}+{v}_{z}^{2}}\rangle$ (**a**) as a function of depth [m] and frequency [Hz], and (**b**) the depth averaged spectrum. (**c**) The time series of the amplitude of 30-h lowpass diurnal tidal elevation, D_1_ = K_1_ + O_1_ [m] at Nakanoshima tidal station (Fig. 1a) is shown in red, and that for semi-diurnal tidal elevation D_2_ = M_2_ + S_2_ + N_2_ is shown in black. The diurnal and semi-diurnal constituents are obtained by harmonic analysis for the hourly tidal elevation record at Nakanoshima tidal station. (**d**) The time series of 30-h lowpass near-inertial shear amplitude $\langle \sqrt{{u}_{z}^{2}+{v}_{z}^{2}}\rangle$ measured at Stn. TK1.

The observed strong turbulence associated with high vertical wavenumber internal wave shear implies active occurrence of PSI near the critical latitude 28.9°N. The moored ADCP observations during our shipboard experiment suggest that the velocity field is dominantly modulated at M_2_ semi-diurnal tidal frequency (Supplementary Fig. S5a,b). In contrast, the moored ADCP vertical shear modulates at subinertial, near-inertial (or K_1_ diurnal), as well as M_2_ semi-diurnal tidal frequencies. However, these M_2_ tidal, near-inertial and subinertial peaks in shear spectra are not clear, especially in upper layers, probably because of the Doppler smearing by the strong Kuroshio flow^27^ (Supplementary Fig. S5). The energy of the observed M_2_ semi-diurnal internal tide could be converted to that of high vertical wavenumber near-inertial waves through PSI. To investigate the possibility of PSI and other mechanisms to generate observed high vertical wavenumber internal wave shear, the long-term moored ADCP data obtained at Stn. TK1 from September 2000 through July 2001 were analyzed. Assuming the energy source of the PSI-induced near-inertial waves is M_2_ internal tide, the amplitude of the PSI-induced near-inertial wave shear should modulate fortnightly with spring-neap tidal cycle^55^, similarly to that of M_2_ internal tides. The harmonic analysis of the tidal elevation at Nakanoshima (29.84°N, 129.85°E, Fig. 1a), suggests that the diurnal tidal amplitude (D_1_ = K_1_ + O_1_) is about the half of that estimated for the semi-diurnal component (D_2_ = M_2_ + S_2_ + N_2_) (Supplementary Fig. S6b). The 30-h lowpass near-inertial shear amplitude exhibits subinertial variations with larger amplitude found in the upper 250 m (Supplementary Fig. S6c). Most of these subinertial variations are found to occur fortnightly, with a period of 14 days, throughout the water column (Fig. 6a,b). The fortnightly modulation found in the near-inertial shear amplitude may imply occurrence of the PSI^55^. However, our observation sites are very close to the critical latitude, where the frequency of diurnal tidal flow is at the near-inertial frequency. It is also possible that diurnal near-inertial internal tides are generated directly from the nearby rough topography, which then propagate to the observation sites. To elucidate whether fortnight modulations of near-inertial shear amplitude are correlated more with those of semi-diurnal D_2_ or diurnal D_1_ tidal amplitude, the correlation analyses between D_2_ or D_1_ tidal amplitude and near-inertial shear amplitude are conducted (Supplementary Fig. S7). The correlation between D_1_ diurnal tidal amplitude and the 30-h lowpass near-inertial shear amplitude is significantly high with a positive Spearman correlation coefficient of 0.59. On the other hand, the correlation with D_2_ semi-diurnal tidal amplitude is low, at 0.05. The results suggest that although the near-inertial shear amplitude modulates at the fortnight frequency, the dominant source of the near-inertial energy is D_1_ diurnal tides, not D_2_ semi-diurnal internal tides through PSI. Our study estimates 10–100 mW m^−2^ for the K_1_ internal tide energy generation rate at the bottom near Tokara Strait, based on a regional numerical simulation^56^ which could be sufficiently large to generate an observed vertical energy flux of the near-inertial internal waves, 0.1–1 mW m^−2^. Accordingly, the local PSI of M_2_ internal tides, with the anomalously low minimum internal wave frequency due to geostrophic shears, is unlikely. However, if the PSI-induced near-inertial internal waves are advected by the Kuroshio from the upstream region south of the critical latitude, the fortnightly modulations of the Doppler-smeared near-inertial shear amplitude should be lagged behind that of the D_2_ semi-diurnal tide. The lagged Spearman correlation coefficients between the D_2_ semi-diurnal tidal amplitude and the 30-h lowpass near-inertial shear amplitude show a maximum correlation coefficient of 0.34 with a 94 h time lag (Supplementary Fig. S7). With a distance from the critical latitude region to the observation sites along the Kuroshio, ~160 km, the time lag of 94 h implies an average advection speed at 0.47 m s^−1^. The estimated advection speed is consistent with the velocity magnitude observed in the subsurface layers of the Kuroshio, ~0.5 m s^−1^ between 200–400 m depth (Fig. 2a,d). Assuming that the estimated near-inertial wave energy, *O*(10 J m^−3^) (see Methods section) is continuously dissipated by the observed bands of the turbulent dissipation rates, *O*(10^−8^–10^−7^ W kg^−1^), with the reference seawater density *ρ*_*o*_ = 1025 kg m^−3^, the internal waves could be dissipated completely after 1–10 days. Therefore, it is possible that the PSI-induced near-inertial waves can survive ~4 days after the 94-h advection. Even without the tide or PSI, near-inertial internal waves of high vertical wavenumber could be generated by the Kuroshio over the topography^19^. The globally estimated energy generation rate from geostrophic flow to lee wave by the study of Nikurashin and Ferrari^19^ in Okinawa Trough, the region near the observation sites, is *O*(0.1–1 mW m^−2^), consistent with the vertical energy flux of observed near-inertial internal waves.

The local generation of the D_1_ diurnal internal tide is the most plausible and likely candidate for the mechanism generating high vertical wavenumber near-inertial internal waves observed near Tokara Strait. The same harmonic analysis for the diurnal and semi-diurnal tidal elevations during the shipboard observations in November 2016 at Tanegashima tidal station (30.47°N, 130.97°E, Fig. 1a) shows a relatively large diurnal tidal amplitude during the UVMP transect observations in Leg A (November 14–15) and Leg B (November 18–19) (Supplementary Fig. S8c). The near-inertial waves induced by the Kuroshio over the topography cannot be ruled out. Other possibilities, such as the spontaneous generation of the near-inertial waves by the meandering Kuroshio^24–28^, and the PSI of wind-induced near-inertial waves^31^ are not discussed in the present study due to the limited observation data. More intensive field campaigns, and high-resolution numerical simulations, are necessary to definitively identify the source of these near-inertial waves propagating in the upstream Kuroshio.

The breaking of the near-inertial waves is influenced by the Kuroshio. This is because the near-inertial waves can be trapped in the region of negative relative vorticity on the less dense side of the Kuroshio and of strong geostrophic vertical shear below the Kuroshio^32–34^. The modulation of the lowest internal wave frequency, as known as the effective Coriolis frequency *f*_*eff*_, by the vertical component of the relative vorticity in the Kuroshio can be equated for a meridional two-dimensional front,

2 $${f}_{eff}=f\sqrt{(f+\frac{\partial v}{\partial x})\,{f}^{-1}},$$

where *v* is the mean meridional velocity and *x* is the zonal coordinate^33^. Considering the contribution from vertical shear of the mean flow to draw down the lowest internal wave frequency further, the equation becomes^34^

3 $${\omega }_{min}=f\sqrt{(f+\frac{\partial v}{\partial x})\,{f}^{-1}-{(\frac{\partial v}{\partial z})}^{2}\,{(\frac{\partial b}{\partial z})}^{-1}}.$$

**Figure 7:**
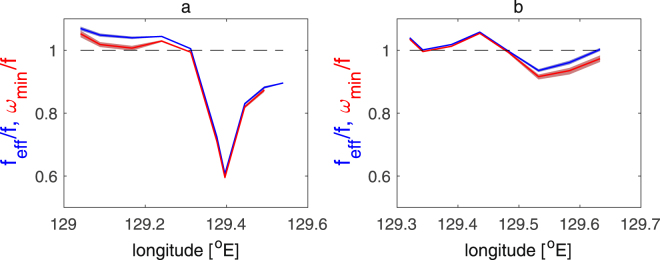
Normalized minimum internal wave frequencies by local Coriolis frequency averaged within the upper 500 m as a function of longitude for (**a**) Leg A and for (**b**) Leg B. Blue indicates the minimum internal wave frequency for a barotropic front *f*_*eff*_/*f* (2)^33^, and red indicates that for a baroclinic front *ω*_*min*_/*f* (3)^34^. Shading represents 95% confidence interval for each average value. Horizontal dashed line indicates unity.

These lowest internal wave frequencies are computed with the measured ADCP velocity and buoyancy along the transect averaged over 200 m depth and 7 km for vertical and horizontal direction, respectively, replacing *v* with the computed velocity component normal to the ship track, *x* by the direction parallel to the ship track, and ${(\frac{\partial v}{\partial z})}^{2}$ by total velocity shear square ${(\frac{\partial u}{\partial z})}^{2}+{(\frac{\partial v}{\partial z})}^{2}$. Because our ship tracks were not normal to the front, the contribution from the vertical relative vorticity to modulate *f*_*eff*_ and *ω*_*min*_ is most likely underestimated. Nevertheless, both the average *f*_*eff*_ and *ω*_*min*_ within the upper 500 m show a similar trend in which the lowest internal wave frequencies are significantly decreased around 129.3–129.5°E in Leg A (Fig. 7a) and 129.5–129.65°E in Leg B (Fig. 7b) from the local Coriolis frequencies *f*. The reduction of the lowest internal-wave frequency and the slightly larger lowest internal-wave frequency formed to the west, create the convex-down structure in *f*_*eff*_ and *ω*_*min*_ at several longitude ranges, where the near-inertial waves can be trapped. In these regions, the amplitudes of high vertical wavenumber shear seems large, especially at 129.3–129.6°E in Leg A (Figs 2b,c and 7a). The large turbulent dissipation rates are found beneath the Kuroshio at 100–150 m depth and on the less dense side of the Kuroshio below 150 m depth at 129.3–129.6°E in Leg A and at 129.5–129.65°E in Leg B (Fig. 5), where the near-inertial wave trapping favorable structures are coincidently formed (Fig. 7). This implies that the trapping mechanisms by the geostrophic shear may be effective in promoting near-inertial internal-wave breaking and causing enhanced turbulence in the upstream Kuroshio.

The induced energy dissipations and vertical mixing could have important implications for watermass modifications. A previous study^57^ estimated the vertical eddy diffusivity at the salinity minimum layer *σ*_*θ*_ = 26.8 kg m^−3^ of North Pacific Intermediate Water (NPIW) in the Okinawa Trough to be 6.8–21.5 × 10^−4^ m^2^ s^−1^ using a steady-state salinity balance with historical current data and salinity data obtained by the profiling floats. Although the direct turbulence data obtained in this study cover only the upper 300 m depth, and did not reach the layer of *σ*_*θ*_ = 26.8 kg m^−3^, strong turbulence just above salinity minimum layer of NPIW, which is equivalent to >*O*(10^−4^ m^2^ s^−1^) of average eddy diffusivity, is consistent with a previous estimate^57^. This implies that the observed large amplitude high vertical wavenumber internal waves play very important roles in watermass mixing and modification in the study region.

Not only is the observed pronounced turbulent diapycnal mixing important for the watermass modifications of NPIW, it is also very important for nutrient supply from the lower layer to the surface euphotic layer. Recent studies of the nutrient transport in the upstream Kuroshio showed that despite its low-nutrient oligotrophic surface water, the Kuroshio transports a large amount of nitrate at an average rate of 170.8 kmol s^−1^ in the subsurface layer, *σ*_*θ*_ = 24–27 kg m^−3^ and transports it downstream^6,7^, similar to the nutrient stream of the Gulf Stream^8–11^. In addition, nitrate concentrations below the Gulf Stream are found to be higher than in the ambient water of the same density^8,9^. This along-isopycnal anomaly of nutrients is most likely formed and maintained by diapycnal diffusion, as opposed to homogenizing mesoscale along-isopycnal stirring. Therefore, large average turbulent diffusivity of *O*(10^−4^ m^2^ s^−1^) in the subsurface layer at 100–300 m depth, *σ*_*θ*_ = 24–26 kg m^−3^, reported by this study, indicates that the observed subsurface turbulence induced by the near-inertial internal waves could effectively diffuse up the subsurface nutrient stream of *σ*_*θ*_ = 24–27 kg m^−3^ to the shallower layer, generate the elevated nutrient concentrations along the Kuroshio, and increase primary production in its downstream.

In this study, for the first time, the spatially coherent banded layers of strong turbulence associated with high vertical wavenumber near-inertial internal waves were directly observed using a new tow-yo microstructure profiler. Our analyses suggest that the high vertical wavenumber near-inertial waves are most likely generated by the diurnal tide over the rough topography in Tokara Strait. Because the observation site is around 30°N latitude, where the inertial period is 24 h, the generated diurnal internal tides are inherently near-inertial waves with slow group velocities. The ADCP and microstructure data suggest that these near-inertial internal waves are trapped and broken into turbulence, preferentially on the anticyclonic side of the upstream Kuroshio, suggesting that the trapping of the near-inertial waves by the Kuroshio due to anticyclonic vorticity^33^ catalyzes the breaking and dissipation of the near-inertial/diurnal internal waves, and promoting watermass modification and nutrient supply. Although the contributions of PSI to form observed high vertical wavenumber near-inertial waves were found to be small compared to the diurnal internal tide in our observations, other mechanisms to generate observed inertial waves are not discussed in detail in this study. Okinawa Trough and Tokara Strait are very peculiar regions because of their strong baroclinic current, the Kuroshio (which can modify the lowest internal wave frequency), flowing through the shallow seamounts and islands, and through the critical latitude for PSI and the boundary where the diurnal period is identical to the inertial period. More comprehensive field and numerical studies are, therefore, necessary to fully understand the generation, propagation, and dissipation mechanisms of these near-inertial internal waves/diurnal internal tides propagating in the upstream Kuroshio.

## Methods

### Back-rotation of the shear

Near-inertial wave velocity and shear rotate clockwise in time in the Northern Hemisphere. When the observation period exceeds or is equivalent to the inertial time scale, this rotation needs to be removed to avoid temporal aliasing of lateral velocity and shear structures. The back-rotated shear *Z*(*t*_0_) = *u*_*z*_(*t*_0_) + *iv*_*z*_(*t*_0_) (where *u*_*z*_ and *v*_*z*_ are the zonal and meridional shear, respectively, and *i* is an imaginary unit) referenced to the time *t*_0_, is estimated from the observations *Z*(*t*) = *u*_*z*_(*t*) + *iv*_*z*_(*t*) at the arbitrary time *t* as

4 $$Z({t}_{0})=Z(t){e}^{i(t-{t}_{0})\bar{f}},$$

where $\bar{f}$ is the mean Coriolis frequency, assuming the rotation at the mean inertial frequency, and back-rotated zonal *u*_*z*_(*t*_0_) and meridional shear *v*_*z*_(*t*_0_) are obtained by taking the real and imaginary parts of (4), respectively.

### Tow-yo turbulence survey

We used a new tow-yo Underway Vertical Microstructure Profiler (Underway-VMP: UVMP) to measure high-resolution spatial structures of microscale turbulence at the Kuroshio Front. The UVMP consists of a vertical microstructure profiler, VMP-250 (Rockland Scientific International, Victoria, Canada) and a winch for the Underway-CTD (UCTD, Teledyne Oceanscience, USA). The VMP-250 carries two shear probes, two FP07 thermistors, a pressure sensor, and a vibration sensor to measure microscale velocity shear, microscale temperature gradient, pressure and accelerations, respectively, at a frequency of 512 Hz. The UVMP was tow-yoed at a ship speed relative to water, at 1.5–2.1 m s^−1^. The descending speeds of the UVMP, gradually decreasing with depth, were between 0.3–0.8 m s^−1^. Taking 7 minutes to reach 300 m depth and 5 minutes for recovery, the lateral resolution of the tow-yo survey was 1–2 km, depending on the absolute current velocity. The data were internally recorded and recovered after each transect observation. However, because the both FP07 thermistors mounted initially on the VMP-250 in Leg A malfunctioned, the expendable bathythermographs (XBT-T7s) were deployed six times to obtain temperature profiles. The XBT temperature data were interpolated by objective mapping with decorrelation lengths of 15 km and 40 m along ship-track and vertical directions, respectively, which were then used to estimate the background potential density *ρ*_*θ*_ and *σ*_*θ*_ and stratification for Leg A (see below). For Leg B, two functional FP07 thermistors were installed on the VMP-250, so that the potential density *ρ*_*θ*_ and *σ*_*θ*_ could be estimated, based on high-resolution FP07 thermistor data. To cross-calibrate the FP07 thermistors, the calibrated UCTD sensor and VMP-250 were clamped together and lowered to 200 m depth with the BT-winch of the *Kagoshima*-*maru*.

To compute turbulent kinetic energy dissipation rates *ε*, the turbulent shear spectra were computed using the microscale shear data measured by the airfoil shear probe over 8 seconds, at approximately every 5–6 m depth, with an overlap of 4 s (2.5–3 m). The obtained shear spectra were integrated over the wavenumber ranges, where spectra agree with the Nasmyth empirical shear spectrum^51^, avoiding the integrations of electronic noise at high wavenumber ranges and low frequency instrument vibration noise at low wavenumber ranges. For the wavenumber ranges where the observed and the empirical spectra did not match, we integrated the fitted Nasmyth empirical shear spectrum^51^ (see Fig. 4). Assuming isotropic turbulence, *ε* was then computed by

5 $$\varepsilon =\frac{15}{2}\nu \,\bar{{(\frac{\partial u}{\partial z})}^{2}},$$

where *ν* is the kinematic viscosity, and $\bar{{(\partial u/\partial z)}^{2}}$ is the turbulent shear variance computed by the integration of the shear spectrum.

Because the UVMP does not carry a conductivity sensor to measure salinity, seawater potential density *ρ*_*θ*_ and *σ*_*θ*_ = *ρ*_*θ*_ − 1000 (kg m^−3^) were estimated using the close relationship between potential temperature and potential density found at the observation sites (Supplementary Fig. S1). The relationship between potential temperature and potential density was derived from the UCTD data obtained across the front prior to the observations in Legs A and B (Fig. 1a), and from CTD data obtained at 36 stations during the surveys (Fig. 1a). The empirical relationship was derived by bin-averaging the observed potential density as a function of potential temperature every 0.5 °C (Supplementary Fig. S1). The potential density *ρ*_*θ*_ and *σ*_*θ*_ were then estimated by linear interpolation with the derived mean density model as a function of potential temperature. The standard deviation of the mean density in each average bin was as small as <0.03 (kg m^−3^) for *σ*_*θ*_ > 25 (deeper than 150 m depth), 0.04–0.1 (kg m^−3^) for 24 < *σ*_*θ*_ < 25 (100–150 m depth), and ~0.12 (kg m^−3^) for *σ*_*θ*_ < 24 (shallower than 100 m depth); the estimated density had better accuracy for the denser and deeper layers.

### Internal wave energy flux and wind power input

The internal wave energy flux along the vertical axis was estimated based on the wave horizontal kinetic energy HKE, and the vertical group velocity *Cg*_*z*_, assuming that the near-inertial waves had low potential energy. Using the dispersion relation of the internal waves, the vertical group velocity for the low frequency internal wave is

6 $$| C{g}_{z}| =| \frac{{f}^{2}\,-\,{\omega }^{2}}{\omega m}| ,$$

where *ω* is the internal wave frequency (rad s^−1^), and *m* the internal-wave vertical wavenumber (rad m^−1^). The horizontal kinetic energy of the internal waves is

7 $${\rm{HKE}}=\frac{1}{2}{\rho }_{o}\frac{[{u}_{z}^{2}+{v}_{z}^{2}]}{{m}^{2}},$$

where *u*_*z*_ and *v*_*z*_ are ADCP zonal and meridional shear (s^−1^), respectively, and [] represents the spatiotemporal average between 100–250 m depth and over the entire transect for Leg A and Leg B. The internal-wave frequency *ω* was estimated as *ω* = 1.01 *f* (rad s^−1^), based on the internal ray path and the measured velocity shear band (Fig. 2) following a previous study^25^. The range of the internal-wave vertical wavelength was estimated as 100–200 m with a mean of 150 m, based on the spectral peaks in the shear spectra (Fig. 3c,d). The corresponding wavenumber range *m* = 0.063–0.031 and its mean *m* = 0.042 rad m^−1^ were used to estimate the group velocity *Cg*_*z*_ and HKE. The estimated horizontal HKE were 2.9–11.5 with a mean of 6.4 J m^−3^ for Leg A and 6.3–25.2 with a mean of 14.2 J m^−3^ for Leg B. The range of the vertical group velocity was estimated to be 2.3–4.6 × 10^−5^ m s^−1^ with a mean of 3.5 × 10^−5^ m s^−1^. The resulting vertical internal wave energy flux for Leg A was 0.07–0.5 mW m^−2^ with a mean of 0.2 mW m^−2^, and that for Leg B was 0.1–1.2 mW m^−2^ with a mean of 0.5 mW m^−2^ (Supplementary Fig. S3b).

To compare the internal wave energy flux with the wind power input to the near-inertial motions, a previous slab model^48^ was used with the hourly reanalysis Japan Meteorological Agency GPV-MSM wind data^58^ from October 28 through November 30, 2016. Considering that the Kuroshio may have a strong advective effect and that remote wind-induced inertial motions could propagate to the observation sites, the hourly wind data were first averaged spatially over the region within 26–36°N and 125–131°E. The mixed layer depth was defined by the buoyancy frequency jump from the surface, and found to be 100 m depth where the large amplitude near-inertial shear was observed. Strong winds occurring before field observations on November 8–12, and during observations on November 15 input energy to near-inertial motions (Supplementary Fig. S3).

### Analyses for the tidal elevation and long term moored ADCP data

Hourly tidal elevation data at Nakanoshima tidal station obtained from the Japan Oceanographic Data Center (JODC) were analyzed from August 31, 2000 through August 14, 2001. Harmonic analysis with 59 tidal constituents was conducted for the tidal elevation record to compare with the 30-h lowpass near-inertial shear amplitude (see below). The results of the harmonic analysis showed that the variance of the dominant three components for semi-diurnal (M_2_, S_2_, and N_2_) and two components for diurnal (K_1_ and O_1_) tidal elevation can explain 99% of the total variance, with the M_2_ tidal component accounting for 67%, S_2_ for 13%, N_2_ for 3%, K_1_ for 10%, and O_1_ for 6%. To obtain the time series of subinertial diurnal and semi-diurnal tidal elevation by the harmonic analysis, the computed diurnal D_1_ = K_1_ + O_1_ and semi-diurnal D_2_ = M_2_ + S_2_ + N_2_ tidal elevation amplitudes were smoothed with a 30-h lowpass filter and compared with the 30-h lowpass near-inertial shear (Fig. 6). The same harmonic analysis was conducted for the tidal elevation data obtained during the shipboard observations in November 2016, at Tanegashima tidal station (Fig. 1a). The variance of total tidal elevation at Tanegashima station is contributed nearly identically from each dominant tidal constituent as in the Nakanoshima tidal data.

To compare the obtained 30-h lowpass semi-diurnal and diurnal tidal amplitudes with the near-inertial shear amplitude, the relatively long-term moored ADCP data were analyzed. The moored ADCP (upward looking 75 kHz 20° beam angle Workhorse Long Ranger ADCP, Teledyne RDI) measured the horizontal current at 14 layers with 32 m vertical bins from 480.5 to 64.5 m depth from September 30, 2000 through July 1, 2001, at Stn. TK1 (Fig. 1a) in the vicinity of the Stn. M01 and M02. The hourly ADCP data were differentiated vertically to obtain the vertical shear. To extract the near-inertial shear, a bandpass Butterworth filter with cutoff frequencies at 0.8 *f* and 1.1 *f* (equivalent to the periods of 30 h and 21.7 h, respectively) was used. The near-inertial amplitude was computed using near-inertial zonal and meridional shear as $\sqrt{{u}_{z}^{2}+{v}_{z}^{2}}$. The subinertial modulations of the near-inertial shear amplitude $\langle \sqrt{{u}_{z}^{2}+{v}_{z}^{2}}\rangle$ was then obtained using a lowpass Butterworth filter at 30 h for the bandpass near-inertial shear amplitude.

### Data Availability

The datasets generated and/or analyzed during the current study are available from the corresponding author upon request.

## Electronic supplementary material

Supplementary Figures
