# Author Correction: First Evidence of Coherent Bands of Strong Turbulent Layers Associated with High-Wavenumber Internal-Wave Shear in the Upstream Kuroshio

**DOI:** 10.1038/s41598-018-26662-4

**Published:** 2018-06-06

**Authors:** Takeyoshi Nagai, Daisuke Hasegawa, Takahiro Tanaka, Hirohiko Nakamura, Eisuke Tsutsumi, Ryuichiro Inoue, Toru Yamashiro

**Affiliations:** 1 Tokyo University of Marine Science and Technology, Department of Ocean Sciences, Tokyo, 108-8477 Japan; 2 Tohoku National Fisheries Research Institute, Japan Fisheries Research and Education Agency, Fisheries Oceanography and Resources Department, Shiogama, Miyagi 985-0001 Japan; 3 Kagoshima University, Faculty of Fisheries, Kagoshima, 890-0056 Japan; 4 Kyushu University, Research Institute for Applied Mechanics, Kasuga, Fukuoka, 816-8580 Japan; 5 Research and Development Center for Global Change, Japan Agency for Marine-Earth Science and Technology, Yokosuka, 237-0061 Japan; 6 Graduate School of Science and Engineering, Kagoshima University, Kagoshima, 890-0065 Japan

Correction to: *Scientific Reports* 10.1038/s41598-017-15167-1, published online 06 November 2017

This Article contains an error in the Acknowledgments section.

‘We thank Capt. Uchiyama, C/O Azuma, Prof. Nishina, Prof. Kobari, Prof. Matsuno, Prof. Senju, Dr. Li, crews, and all the participants of the R/T/V Kagoshima-maru cruise KG1616. T.N. thanks Hancyk, Dr. Wolk at RSI, Dr. Li, Seki, and Yoshii at JAC, Dr. Kokubu at NORTEK, Rutka at STS, support from OMIX (MEXT KAKENHI JP16H01590) and SKED (“Study of Kuroshio Ecosystem Dynamics for Sustainable Fisheries” funded to FRA from MEXT). D.H., T.T., and R.I. thank OMIX (MEXT KAKENHI) JP15H05818. H.N., and E.T. thank OMIX (MEXT KAKENHI) JP15H05821.’

should read:

‘We thank Capt. Uchiyama, C/O Azuma, Prof. Nishina, Prof. Kobari, Prof. Matsuno, Prof. Senju, Dr. Li, crews, and all the participants of the R/T/V Kagoshima-maru cruise KG1616. T.N. thanks Hancyk, Dr. Wolk at RSI, Dr. Li, Seki, and Yoshii at JAC, Dr. Kokubu at NORTEK, Rutka at STS, support from OMIX (MEXT KAKENHI JP16H01590). D.H., T.T., and R.I. thank OMIX (MEXT KAKENHI) JP15H05818. H.N., and E.T. thank OMIX (MEXT KAKENHI) JP15H05821.’

