# Supplementary material for: First Evidence of Coherent Bands of Strong Turbulent Layers Associated with High-Wavenumber Internal-Wave Shear in the Upstream Kuroshio
Source: Sci Rep. 2017 Nov 6;7:14555. doi: 10.1038/s41598-017-15167-1 (PMC5674074; doi:10.1038/s41598-017-15167-1)
Supplement: Supplementary file 1 — Supplementary Figures [file 41598_2017_15167_MOESM1_ESM.pdf]

Supplementary information for ”First Evidence of Coherent Bands of Strong Turbulent Layers Associated with High-Wavenumber Internal-Wave Shear in the upstream Kuroshio”

Takeyoshi Nagai[1,*], **Daisuke Hasegawa**[2,+], **Takahiro Tanaka**[2,+], **Hirohiko Nakamura**[3,+], **Eisuke Tsutsumi**[4,+], **Ryuichiro Inoue**[5,+], **and Toru Yamashiro**[6,+]

[1]Tokyo University of Marine Science and Technology, Department of Ocean Sciences, Tokyo, 108-8477, Japan
[2]Tohoku National Fisheries Research Institute, Japan Fisheries Research and Education Agency, Fisheries Oceanography and Resources Department, Shiogama Miyagi, 985-0001, Japan
[3]Kagoshima University, Faculty of Fisheries, Kagoshima, 890-0056, Japan
[4]Kyushu University, Research Institute for Applied Mechanics, Kasuga Fukuoka, 816-8580, Japan
[5]Research and Development Center for Global Change, Japan Agency for Marine-Earth Science and Technology, Yokosuka, 237-0061, Japan.
[6]Graduate School of Science and Engineering, Kagoshima University, Kagoshima, 890-0065, Japan
[*]tnagai@kaiyodai.ac.jp
[+]these authors contributed equally to this work

ABSTRACT

This supplementary information includes figures for (Figure S1) the density model as a function of potential temperature used in this study, for (Figure S2) the Richardson number, for (Figure S3) wind power input to inertial motions and internal wave energy flux, for (Figure S4) the comparison between observed dissipation rates and the previous internal-wave parameterization, for (Figure S5) shear spectra of horizontal velocity measured by the moored upward-looking ADCP, for (Figure S6 and S7) long term moored ADCP data analyses, and for (Figure S8) harmonic analysis for the tidal elevation data collected during the shipboard observations in November 2016.

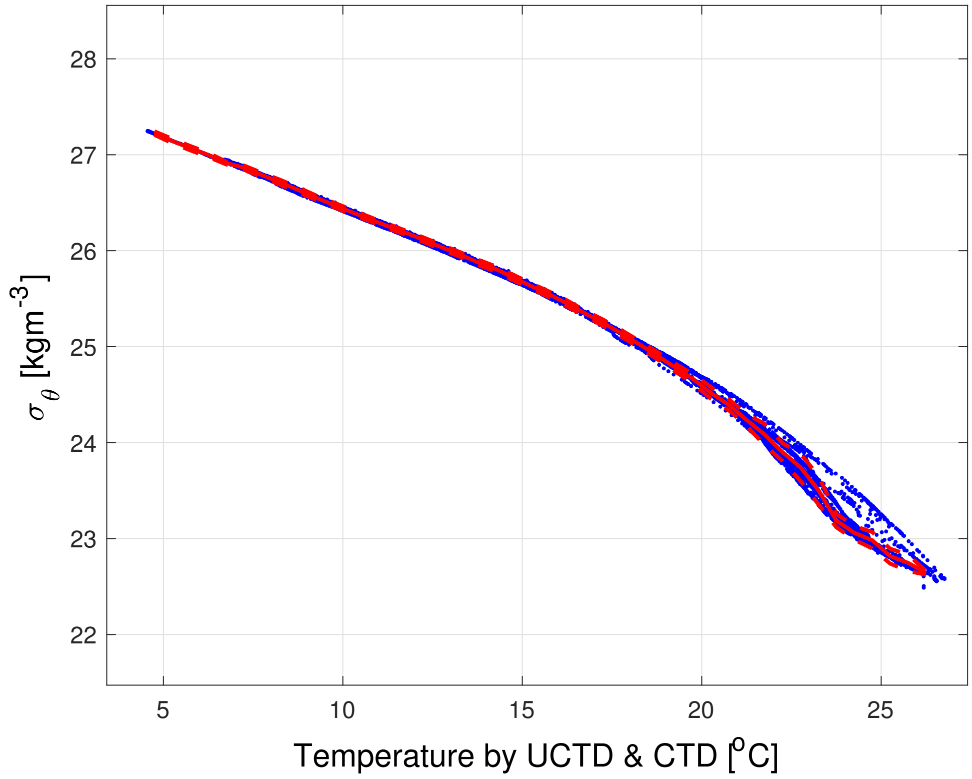

Figure S 1. Potential density σ_θ measured by Conductivity-Temperature-Depth (CTD) and Underway-CTD (UCTD) as a function of potential temperature θ is shown by blue dots. UCTD data are cross-calibrated against the calibrated CTD data. Bin-averaged potential density every 0.5 °C and its standard deviation are shown as red solid and dashed lines, respectively.

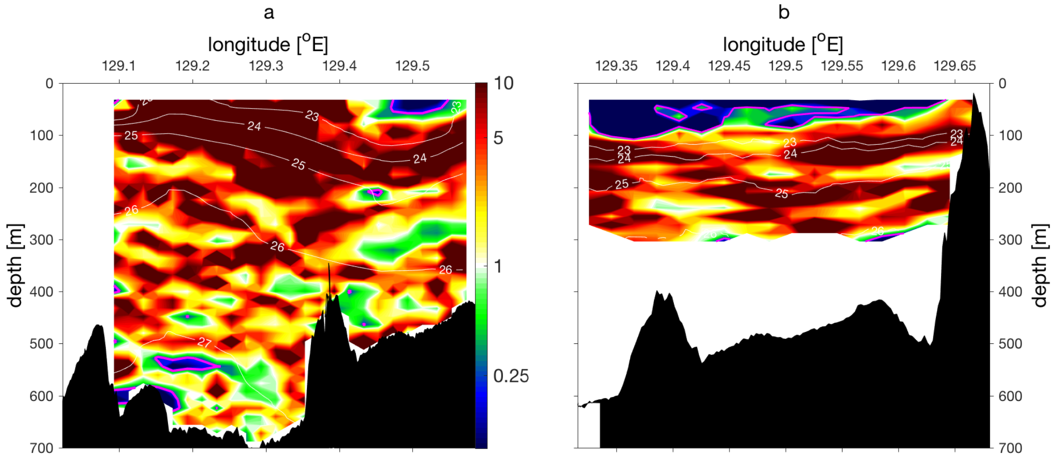

Figure S 2. Richardson number Ri calculated from ADCP shear and buoyancy frequency for (a) Leg A and (b) Leg B. Magenta contours indicate the critical value for K-H instability, $Ri = 0.25$, and white contours are σ_θ. Color is in log scale.

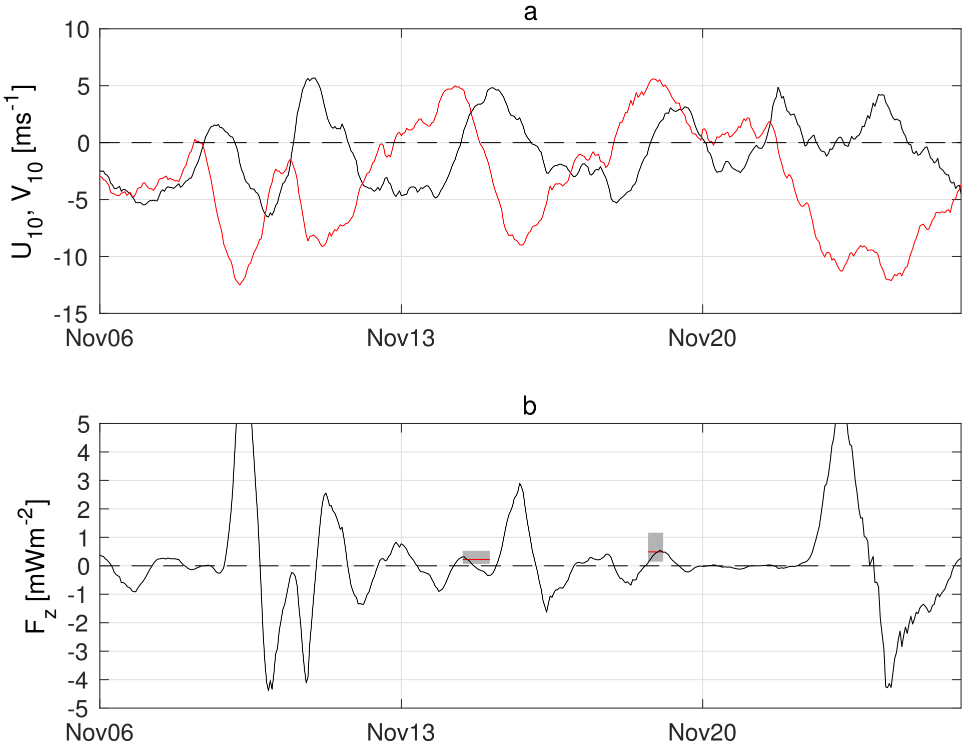

Figure S 3. Wind energy input and internal wave energy flux. (a) Hourly reanalysis of GPV-MSM (Grid Point Values-mesoscale non-hydrostatic model) winds (Japan Meteorological Agency) averaged over the region within 26-36°N and 125-131°E for (black) zonal and (red) meridional wind [m s^{-1}]. (b) Wind energy flux into inertial motions estimated using a slab model with the hourly GPV winds. The estimated internal-wave vertical energy flux is shown in red for Leg A (Nov. 14) and Leg B (Nov. 18). Shading indicates the lower and upper bound with the internal-wave vertical wavenumber range considered $m = 0.063 - 0.031$ and its mean $m = 0.042$ (rad m^{-1}) .

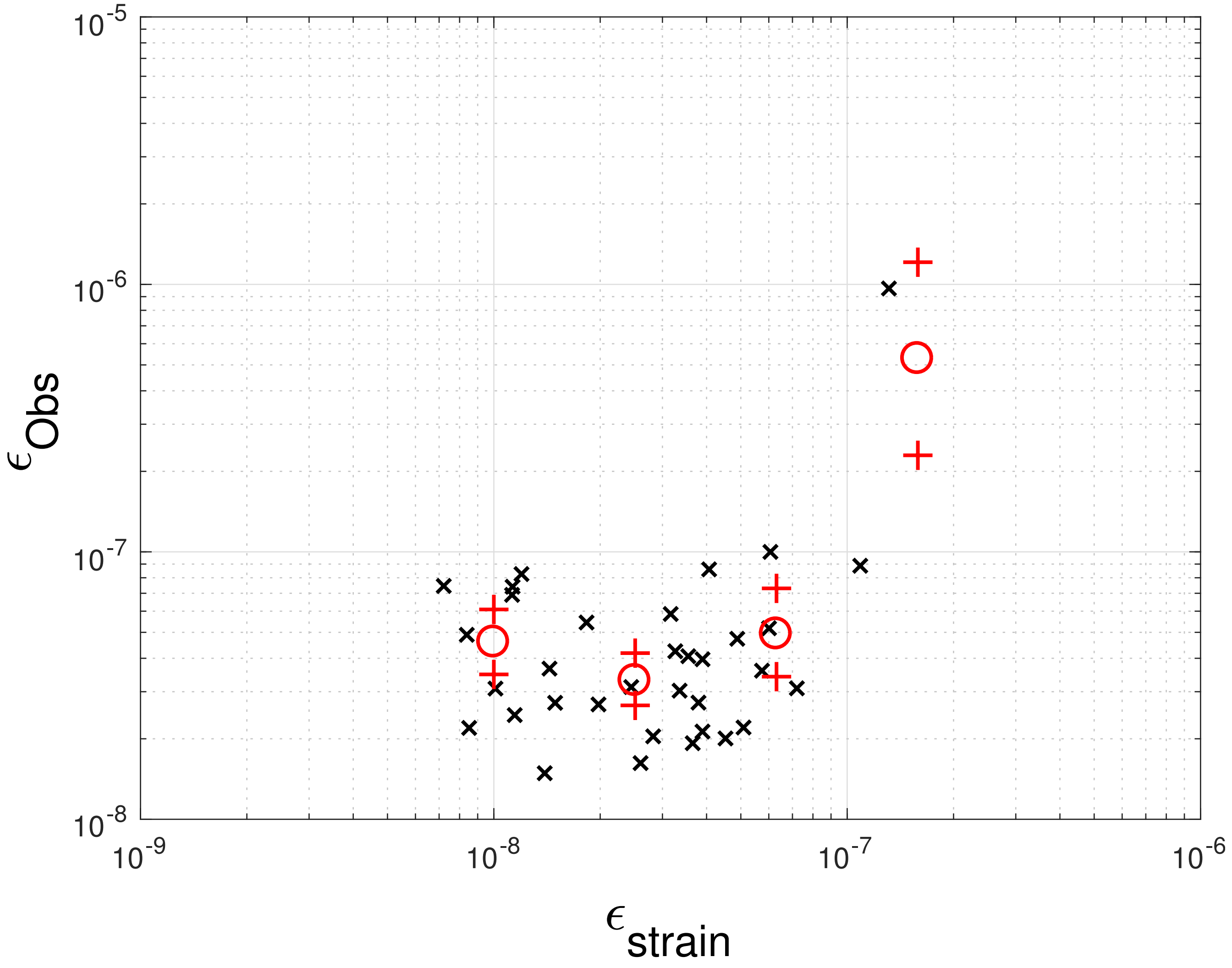

Figure S 4. Comparison between measured turbulent kinetic energy dissipation rates ε_{Obs} [W kg^{-1}] and the ones estimated with the internal wave strain based parametrization ε_{strain} (See Methods section). Each dissipation rate sample is indicated by black crosses. Red circles are bin-average observed dissipation rates in log-space with 95% confidence interval for each average.

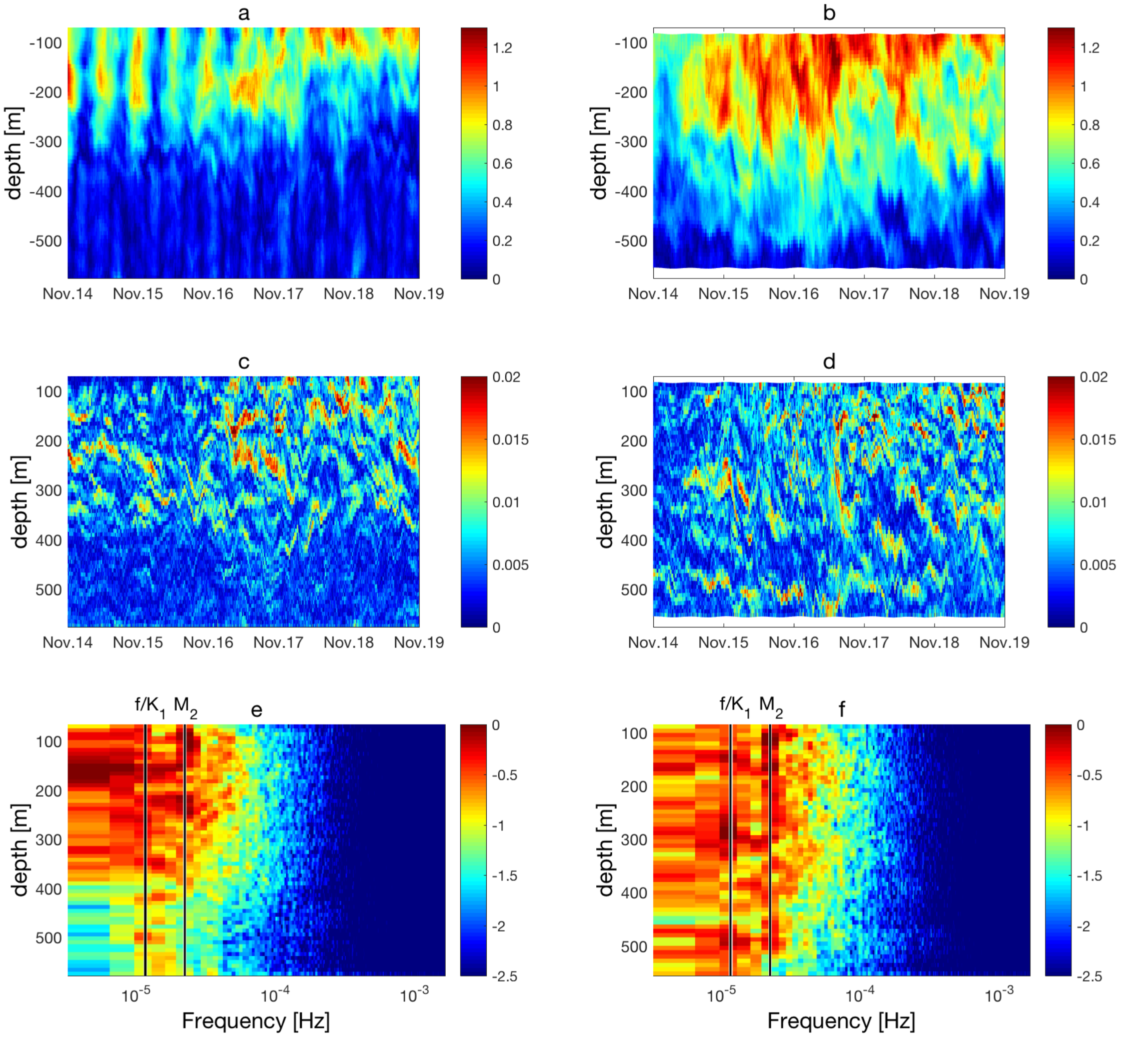

Figure S 5. Moored ADCP measurements at (a, c, e) Stn. M01 and (b, d, f) M02 (Figure 1a) during November 13-19, 2016 for (a-b) current velocity magnitude (m s^{-1}), for (c-d) vertical shear (s^{-1}), and for (e-f) frequency spectra of vertical shear as a function of depth and frequency $\log_{10}$ (s^{-2} Hz^{-1}). Black vertical lines indicate inertial f or diurnal tidal K_1, and M_2 semi-diurnal tidal frequencies.

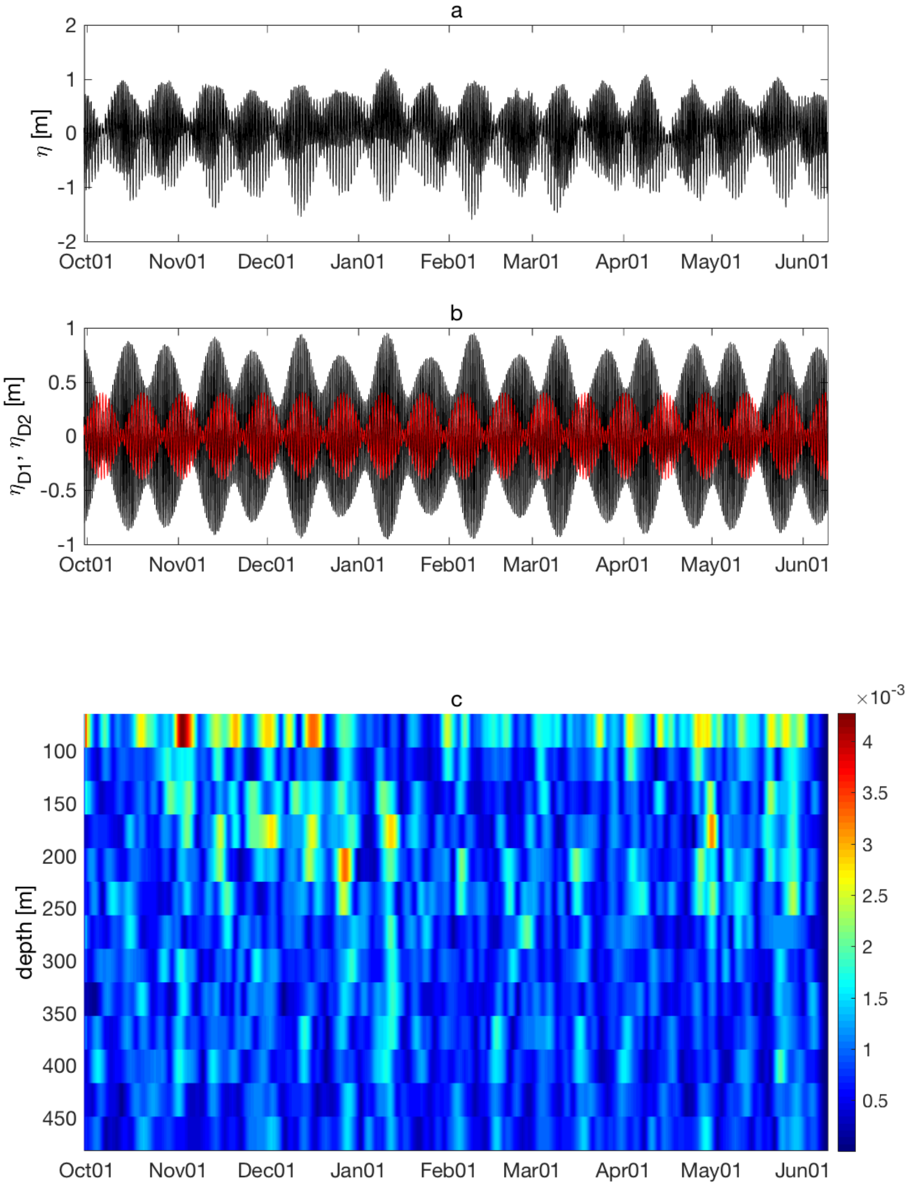

Figure S 6. Long-term moored ADCP measurements from September 30, 2000 through July 1, 2001 at Stn. TK1 and tidal record. (a) Tidal elevation [m] at Nakanoshima tidal station (Figure 1a) from October, 2000 through June, 2001. (b) The semi-diurnal tidal amplitude [m], $D_2=M_2+S_2+N_2$ obtained by the harmonic analysis is shown in black, and that for the diurnal amplitude [m], $D_1=K_1+O_1$ is shown as red. (c) The 30-h lowpass near-inertial shear amplitude [s^{-1}] measured by moored ADCP at Stn. TK1 as a function of time and depth.

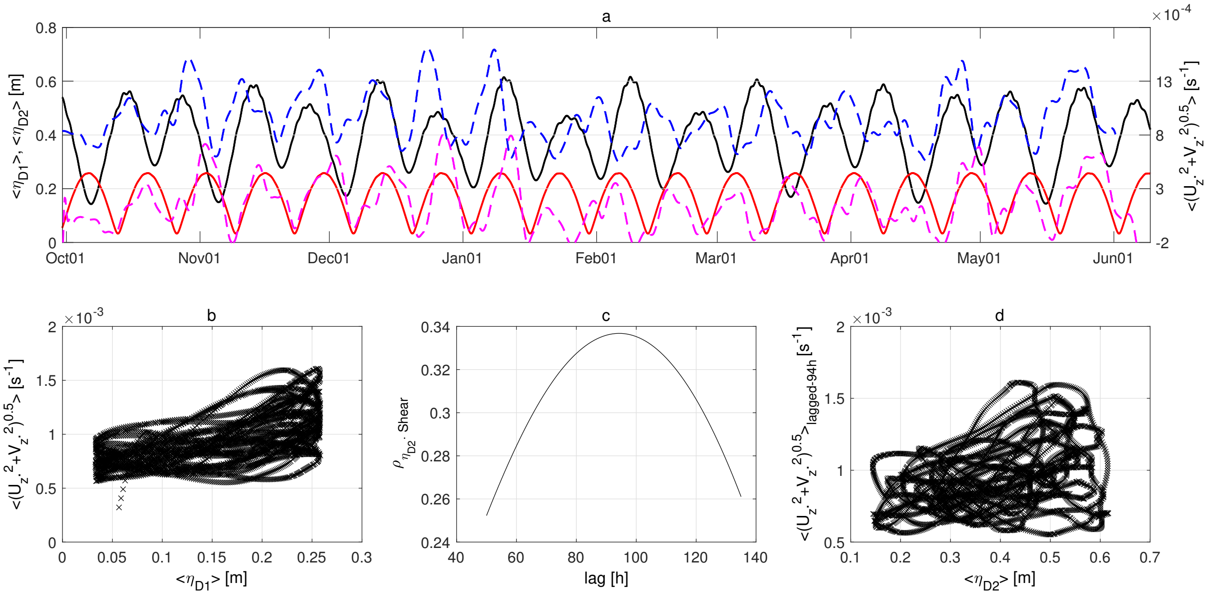

Figure S 7. The correlation between the 30-h lowpass amplitude of semi-diurnal and diurnal tidal elevations and the 30-h lowpass near-inertial shear. (a) The 30-h lowpass tidal elevation amplitude for (solid black) semi-diurnal and (solid red) diurnal components are compared with the 30-h lowpass near-inertial shear (dashed magenta) with zero time lag and (dashed blue) with a 94 h time lag. (b) Scatter plot of the 30-h lowpass amplitude of diurnal tidal elevations and the 30-h lowpass near-inertial shear. (c) Lagged Spearman correlation coefficients between the 30-h lowpass amplitude of semi-diurnal tidal elevations and the 30-h lowpass near-inertial shear. (d) Scatter plot of the 30-h lowpass amplitude of semi-diurnal tidal elevations with a 94 h time lag and the 30-h lowpass near-inertial shear.

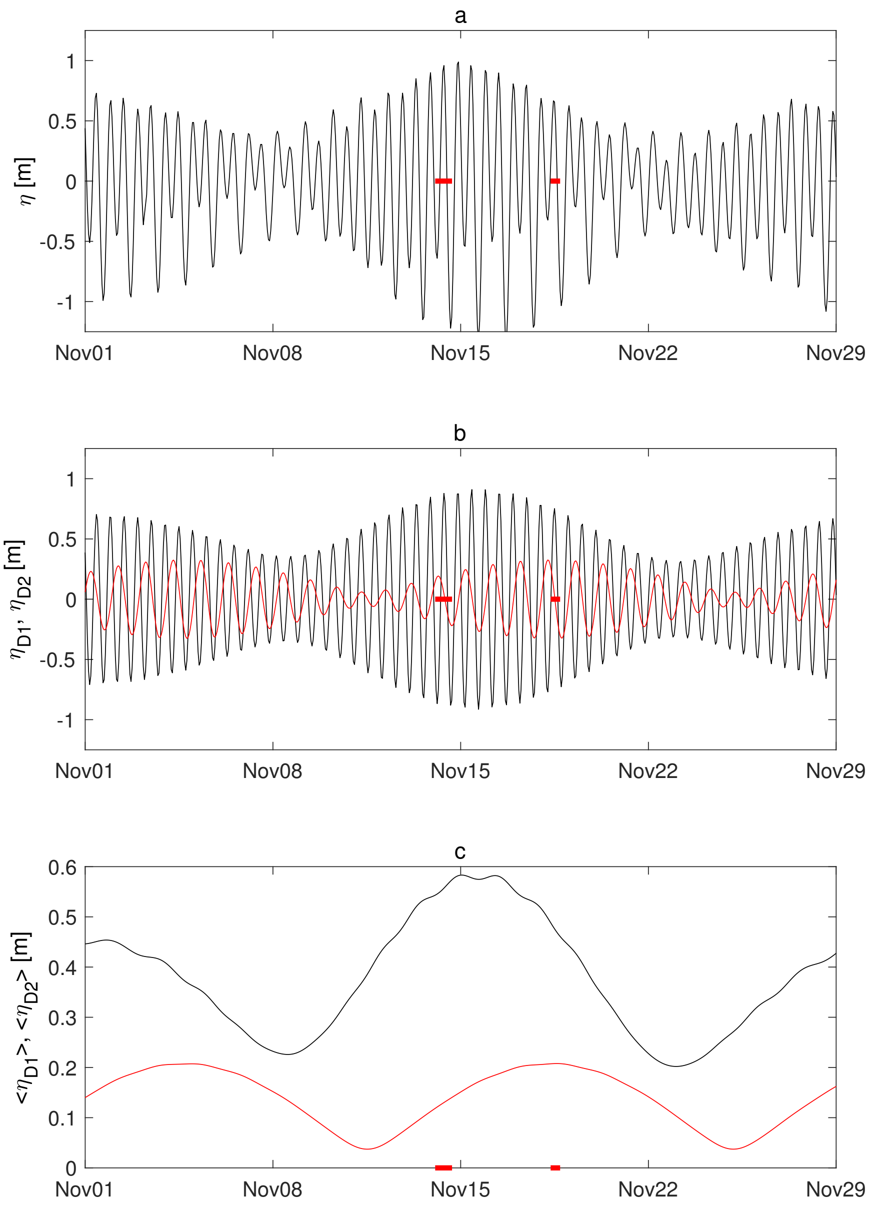

Figure S 8. Tidal elevation and harmonic analysis during shipboard observations in November 2016. (a) Tidal elevation [m] at Tanegashima tidal station (Figure 1a) from November 1, 2016 through November 29, 2016. (b) The semi-diurnal tidal amplitude [m], $D_2 = M_2 + S_2 + N_2$ obtained by the harmonic analysis is shown in black, and that for the diurnal amplitude [m], $D_1 = K_1 + O_1$ is shown as red. (c) The 30-h lowpass semi-diurnal and diurnal tidal amplitudes are shown in black and red, respectively. The periods of observations in Legs A and B are indicated as thick red horizontal lines.
